# Extraction and Analysis of Strontium in Water Sample Using a Sr^**2+**^ Selective Polymer as the Absorbent Phase

**DOI:** 10.1155/2015/425084

**Published:** 2015-11-12

**Authors:** Rongjian Ying

**Affiliations:** College of Chemistry and Chemical Engineering, Linyi University, Linyi 276005, China

## Abstract

A kind of Sr^2+^ selective resin was applied as an absorption phase to extract Sr^2+^ ion from an aqueous solution, and the amount of Sr^2+^ was determined using inductively coupled plasma optical emission spectrometer. Factors, including absorption time, temperature, stirring rate, salt-out effect, desorption, and the pH of the aqueous solution, were investigated to optimize the absorption efficiency of Sr^2+^. Foreign ions were examined to observe their effects on the absorption behavior of Sr^2+^. The optimum condition was absorption time at 20 min, pH of aqueous solution 7, temperature of 35°C, and 600 rpm stirring rate. A 10 mL solution of 0.1 mol/L HCl is used as the desorption agent. The linear range of Sr^2+^ concentrations from 50 to 1200 *μ*g/L was investigated with the slope of 183 *μ*g/L. The limit of detection was 21 *μ*g/L with 4.23% relative standard deviation. The correlation coefficient was found to be 0.9947. Under the optimized conditions, the concentrations of Sr^2+^ in four water samples were detected by the developed method. We propose that this method effectively extracts strontium ion from environmental water samples.

## 1. Introduction

Strontium and its isotopes as effective tracers are applied for characterizing geochemical and biogeochemical behaviors in the fields of archaeology [1, 2], water-rock interactions [3], and geologic chronology [4] to identify and trace the origins and evolutions of the climate and the environment [5, 6]. The separation of strontium from alkali and alkaline-earth elements is important for the determination of strontium isotopic composition in natural sample and for the isotopic detection of ^87^Sr and ^87^Rb by thermal ionization mass spectrometry (TIMS) or multicollector inductively coupled plasma mass spectrometry.

Commonly, natural strontium compounds coexist with other alkali and alkaline-earth compounds, such as sodium, calcium, magnesium, and barium compounds, which make the separation of strontium more complex. Fuming nitric acid has been used previously to separate strontium from large quantities of alkali, alkaline-earth, and other elements effectively [7–10].

Recently, the methods for strontium ion separation have been developed to avoid employing toxic fuming nitric acid to separate strontium, a method previously developed by Grahek et al. [11]. The methods include the use of ion-exchange resins [12, 13], dispersive liquid phase microextraction [14, 15], and strontium specific resins [16]. By these methods, strontium ion was separated along with other alkaline-earth elements that demonstrate similar chemical behaviors [7]. Furthermore, alkali elements, such as sodium, potassium, rubidium, and others, interfered with the strontium separation.

In recent years, ion imprinting has been considered as a convenient and powerful method for synthesizing polymers in the presence of desired template ions [17, 18]. Silica gel is used as a support for ion imprinting due to its chemical, mechanical, and thermal stability and low cost [7–10]. The objective of this study is to utilize a Sr^2+^ selective polymer in the presence of silica gel to separate Sr^2+^ in water sample. In the following experiments, the Sr^2+^ selective polymer is used as an absorbent phase for the extraction of Sr^2+^, and the absorbent behaviors of the Sr^2+^ selective polymer are investigated. The factors influencing the efficiency of Sr^2+^ ion extraction, such as absorption time, pH in the aqueous solution, temperature, stirring rate, salt-out effect, and desorption solution, were optimized. In the optimum conditions, the characteristics of the described method were evaluated, and four real water samples were utilized to assess the applicability and reliability of the proposed method.

## 2. Material and Methods

### 2.1. Instruments

An inductively coupled plasma optical emission spectrometer (ICP-OES, Vista MPX, Varian, USA) with a 40 MHz radio frequency generator and a charge-coupled device detector (Vista Chip) was used to detect strontium ion. The spectrometer was operated in a transient signal acquisition mode [15]. An axial-viewing quartz torch, a cyclonic spray chamber, a glass concentric nebulizer, and a peristaltic pump were used. The measurements were carried out at a radio frequency generator power of 1.0 kW, plasma gas flow rate of  15.0 L/min, auxiliary gas flow rate of 1.5 L/min, nebulizer gas pressure of 200 kPa, replicate read time of 5 s, and pump rate of 15 rpm.

### 2.2. Reagents and Materials

Strontium chloride, nitric acid, sodium hydroxide, hydrochloric acid, and sodium hydroxide were analytical grade reagents. A strontium standard solution of 100 *μ*g/mL was purchased from the National Research Center for Certified Reference Materials (Beijing, China). Stock and working standards were prepared with deionized water.

### 2.3. Sr^2+^ Absorption Experiments

To prepare a sample, 5 g of wet gel particles was dispersed in 10 mL SrCl_2_ solution (5 mmol/L). HCl and NaOH solutions were used to adjust pH of the solution, and NaCl solution was used to examine the salt-out effect on the absorption efficiency of Sr^2+^ in the aqueous solution. The concentration of Sr^2+^ in the aqueous phase was determined by ICP-OES.

### 2.4. Selective Recognition Experiments

The absorption experiments were performed in a magnetically heated water bath at a desired temperature. The amount of Sr^2+^ absorbed by the particle (*Q*, in mg per g of dried particle) was calculated by a mass balance relationship, expressed as in

(1) $$\begin{array}{c} Q=\frac{V\left(C_{0}-C\right)}{W}. \end{array}$$

Here, *C*_0_ and *C* are the Sr^2+^ concentrations in the solution before and after absorption, respectively. *V* is the volume of the solution (mL), and *W* is the amount of the absorbent (g). Ions K^+^, Ca^2+^, Ba^2+^, Rb^+^, Cs^+^, and Mg^2+^ were utilized as competitive ions at a concentration of 5.0 mg/mL to determine the selective recognition of Sr^2+^.

## 3. Results and Discussion

In this study, a Sr^2+^ selective polymer was used as absorbent substrate to extract Sr^2+^ from the aqueous phase. To optimize the absorption conditions for Sr^2+^ recovery, the effects of pH, absorption time, stirring rate, temperature, and salt concentration were studied. A series of designed experiments was performed.

### 3.1. Effect of Absorption Time on the Efficiency of Sr^2+^

**Figure 1:**
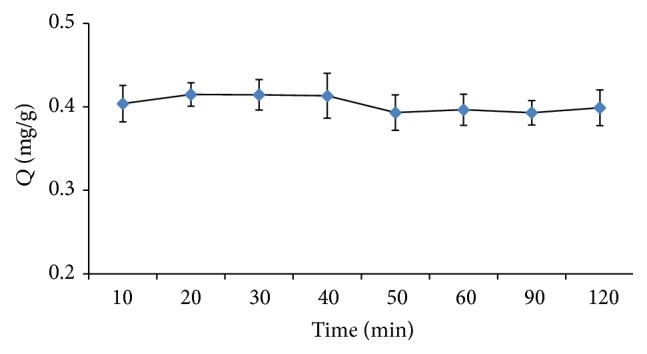
Effect of absorption time on the extraction efficiency (*Q*) of Sr^2+^. Extraction conditions: pH 7, 35°C, and stirring rate 600 rpm.

The absorption time critically affects the efficiency of Sr^2+^ absorption in the aqueous phase by the polymer. In this work, a time range of 10 to 120 min was investigated while holding other parameters constant. It can be seen from Figure 1 that with the change of time to 20 min, the absorbed amount of Sr^2+^ levels off, implying that the absorption equilibrium was reached. The time for absorption equilibrium was fixed at 20 min for all experiments.

### 3.2. Effect of pH on Absorption Quantity of Sr^2+^

**Figure 2:**
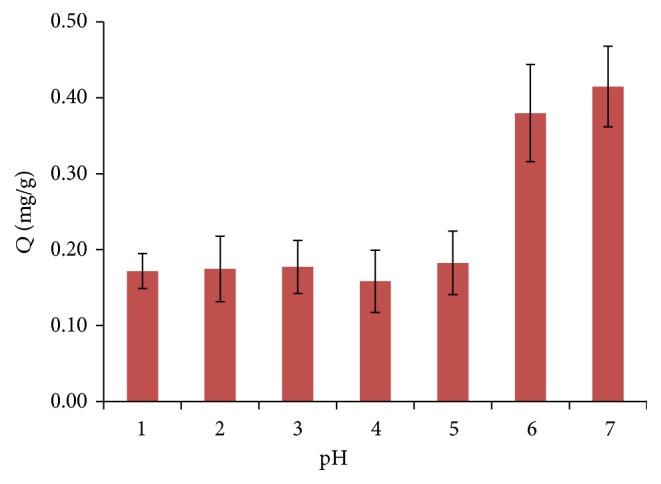
Effect of pH on the extraction efficiency (*Q*) of Sr^2+^. Extraction conditions: absorption time 20 min, 35°C, and stirring rate 600 rpm.

Solution pH has been found to influence complex formation of metal ions and the hydrolysis of cations [19], so pH was varied from 1 to 7 to test the absorption efficiency of Sr^2+^. When the pH ranges from 8 to 14, some ions would precipitate out of solution; thus, basic solutions were not considered for tests. The pH was adjusted with a solution of HCl solution. In Figure 2, it shows that the maximum efficiency for Sr^2+^ absorption is achieved at pH 6 and 7. At low pH, efficiency is poor because of hydrogen bonding with the polymer, which reduces the bonding availability for strontium ions. For subsequent experiments, a pH of 7 was employed.

### 3.3. Effect of Temperature on Absorption Quantity of Sr^2+^

**Figure 3:**
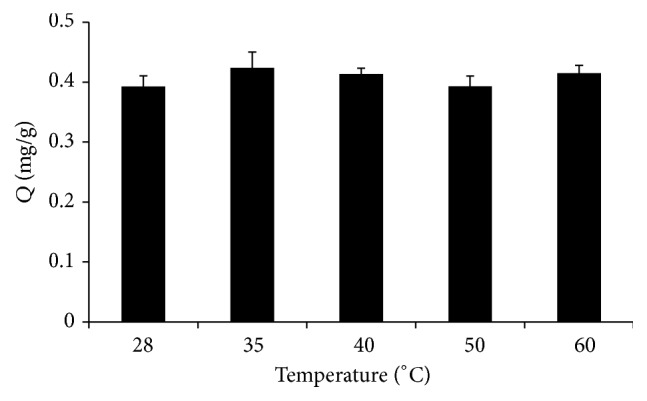
Effect of temperature on the extraction efficiency (*Q*) of Sr^2+^. Extraction conditions: absorption time 20 min, pH 7, and stirring rate 600 rpm.

During extraction, temperature has an important impact on the absorption efficiency of the target in aqueous solution. At high temperature, the diffusion coefficient of strontium ion in aqueous solution is higher, and the absorption time may be shortened [20]. The effect of temperature was studied by evaluating absorption temperature at 28, 35, 40, 45, 50, and 60°C.  Figure 3 shows minimal differences in absorption at the temperatures studied. Although the absorption rate is accelerated with increasing temperature, the partition coefficient depends on temperature, so the effect of temperature on absorption efficiency is not significant. Thus, 35°C was chosen for the subsequent experiments.

### 3.4. Effect of Stirring Rate on Absorption Quantity of Sr^2+^

**Figure 4:**
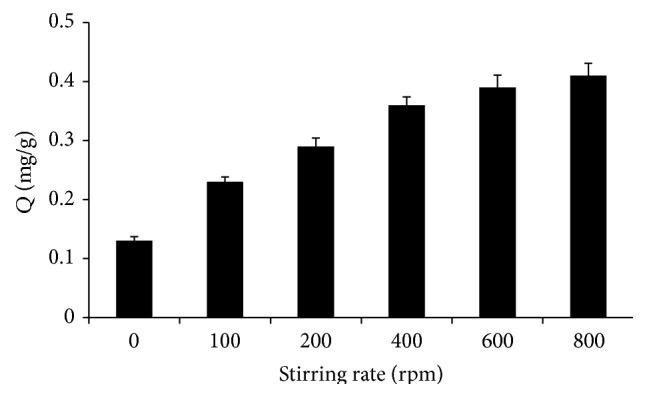
Effect of stirring rate on the extraction efficiency (*Q*) of Sr^2+^. Extraction conditions: absorption time 20 min, pH 7, and 35°C.

During absorption, polymer precipitates out of solution, reducing the absorption efficiency and the interaction between the polymer and the aqueous solution. Magnetic stirring was considered for the method tests. A stirring rate, ranging from 0 to 800 rpm, was taken into consideration.  Figure 4 suggests that the quantity of absorbed Sr^2+^ saturated at stirring rate of more than 600 rpm. A stirring rate of 600 rpm was chosen for the next works.

### 3.5. Salt-Out Effect on Absorption Quantity of Sr^2+^

**Figure 5:**
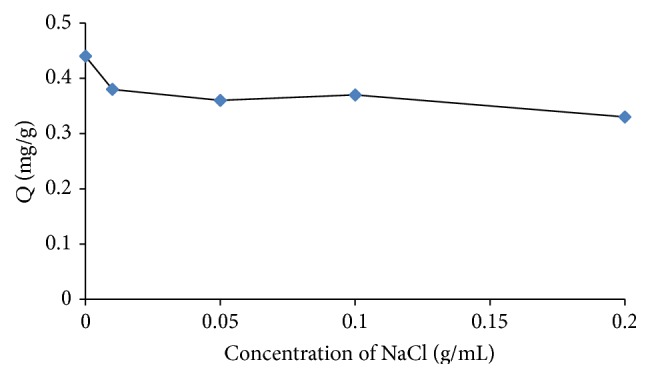
Effect of NaCl concentration on the extraction efficiency (*Q*) of Sr^2+^. Extraction conditions: absorption time 20 min, pH 7, 35°C, and stirring rate 600 rpm.

Salt-out effects can also influence solubility in aqueous solutions, and NaCl solution was used to adjust the ionic strength of the aqueous solution. NaCl was added in concentrations ranging from 0 to 0.2 g/mL. It can be seen from Figure 5 that the Sr^2+^ absorption efficiency is not sensitive to salt-out effects at NaCl concentrations in this range. As NaCl concentration increases, the ion strength of the aqueous solution increases, while the presence of Na^+^ ion reduces the ability of the polymer to form a complex with Sr^2+^ ion. Thus, NaCl was not used to adjust the ionic strength of subsequent solutions.

### 3.6. Desorption of Sr^2+^

**Figure 6:**
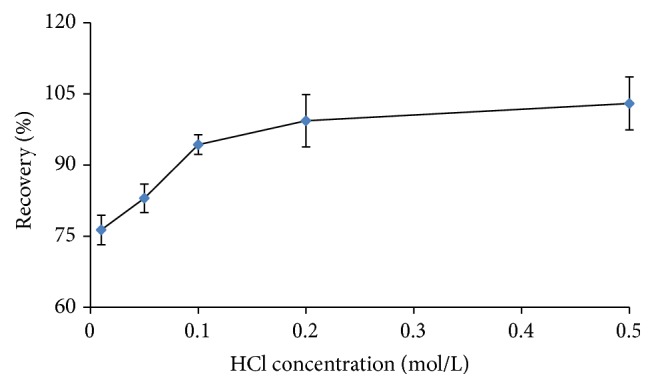
Effect of HCl concentration on the extraction recovery of Sr^2+^.

After completing the absorption of Sr^2+^ ion, desorption of Sr^2+^ was studied using HCl solution as desorption agent. HCl solutions (10 mL) of various concentrations from 0.01 to 0.5 mol/L were used to rinse the polymer resin to investigate the absorption amount of Sr^2+^ at room temperature, as shown in Figure 6. Ten milliliter of 0.1 mol/L HCl solution was sufficient to rinse Sr^2+^ ion out of the particles completely.

### 3.7. Ion Interference

Foreign ions, such as K^+^, Ca^2+^, Ba^2+^, Rb^+^, Cs^+^, and Mg^2+^ ions, can interfere with the absorption of Sr^2+^ in aqueous solution when using the described method. To investigate the effects of foreign ions, a 10 mL solution of 100 *μ*g/L Sr^2+^ mixed with these interfering ions was treated according to the procedure mentioned above. Tolerance limits for interfering ions were established as the highest concentrations that allowed strontium recovery to remain in excess of 90%. The results show that Ca^2+^, Mg^2+^, and Ba^2+^ could be tolerated up to concentrations of 85 *μ*g/L, while K^+^ and Rb^+^ could be tolerated up to a concentration of 100 *μ*g/L. The 85 *μ*g/L of Cs^+^ had no significant effect on the extraction of Sr^2+^.

### 3.8. Analytical Performance

Under optimal conditions, characteristics for the extraction of Sr^2+^ by the method discussed above were obtained. The linear range of absorbed amount of Sr(II) by the polymer was between 50 and 1200 *μ*g/L, and the limit of detection of this method is 21 *μ*g/L with 4.23% of relative standard deviation. The method characteristics are shown in Table 1.

### 3.9. Analysis of Water Samples

Four environmental water samples were used to assess the applicability and reliability of the proposed method. The underground water sample was taken from a local well, tap water was collected from our lab, and river water samples were collected from the Yi River, in Linyi, China, at two locations. Sr^2+^ ions in these samples were extracted and analyzed by the above method and ICP-OES. 50 mL of each water sample was spiked with a 100 *μ*g/L of Sr^2+^ standard solution. The results are listed in Table 2, which indicate that in all cases the recovery of Sr^2+^ from water can be quantitative.

## 4. Conclusions

In this study, a strontium selective polymer was used to extract Sr^2+^ in water sample, and the efficiency was quantified with ICP-OES. The experiment conditions and foreign ions influencing the absorption efficiency of Sr^2+^ on the polymer were investigated. The linear range and limit of detection of the present method are sufficient for the detection of strontium ion. Four real water samples were utilized to evaluate the applicability and reliability of the proposed method. The results suggest that the method will be useful for the extraction and quantification of strontium ion in the environmental samples.

## Figures and Tables

**Table 1:** Linear range, limit of detection, and repeatability of the method.

Analytical parameter	Analytical feature
Linear range (*μ*g/L)	50–1200
Slope (*μ*g/L)	183
Correlation coefficient	0.9947
Limit of detection (*μ*g/L)	21
Relative standard deviation (%)	4.23

**Table 2:** Experimental recoveries of Sr^2+^ in four water samples.

Water samples	Concentration	Added amount	Recoveries
	(*μ*g/L)	(*μ*g/L)	(%)
Underground water	78.2	100	102.3
Tap water	55.8	100	97.7
River water (point 1)	158.4	100	103.9
River water (point 2)	139.6	100	96.4

## References

[1] Bentley R. A. (2006). Strontium isotopes from the earth to the archaeological skeleton: a review. *Journal of Archaeological Method and Theory*.

[2] Frei K. M., Frei R. (2011). The geographic distribution of strontium isotopes in Danish surface waters—a base for provenance studies in archaeology, hydrology and agriculture. *Applied Geochemistry*.

[3] Vengosh A., Spivack A. J., Artzi Y., Ayalon A. (1999). Geochemical and boron, strontium, and oxygen isotopic constraints on the origin of the salinity in groundwater from the Mediterranean coast of Israel. *Water Resources Research*.

[4] Newell D. L., Larson T. E., Perkins G. (2014). Tracing CO_2_ leakage into groundwater using carbon and strontium isotopes during a controlled CO_2_ release field test. *International Journal of Greenhouse Gas Control*.

[5] Price T. D., Burton J. H., Fullagar P. D., Wright L. E., Buikstra J. E., Tiesler V. (2015). Strontium isotopes and the study of human mobility among the ancient maya. *Archaeology and Bioarchaeology of Population Movement among the Prehispanic Maya*.

[6] Trincherini P. R., Baffi C., Barbero P., Pizzoglio E., Spalla S. (2014). Precise determination of strontium isotope ratios by TIMS to authenticate tomato geographical origin. *Food Chemistry*.

[7] Li Q., Liu H. N., Liu T. Y. (2010). Strontium and calcium ion adsorption by molecularly imprinted hybrid gel. *Chemical Engineering Journal*.

[8] Pan J. M., Zou X. H., Yan Y. S. (2010). An ion-imprinted polymer based on palygorskite as a sacrificial support for selective removal of strontium(II). *Applied Clay Science*.

[9] Zhang L., Wei J. Y., Zhao X., Li F. Z., Jiang F., Zhang M. (2015). Strontium(II) adsorption on Sb(III)/Sb_2_O_5_. *Chemical Engineering Journal*.

[10] Zhang Z. L., Li L. (2015). Synthesis and characterization of whisker surface imprinted polymer and selective solid-phase extraction of trace Sr(II) from environment aqueous solution. *Desalination and Water Treatment*.

[11] Grahek Ž., Eškinja I., Košutic K., Lulic S., Kvastek K. (1999). Isolation of radioactive strontium from natural samples: separation of strontium from alkaline and alkaline earth elements by means of mixed solvent anion exchange. *Analytica Chimica Acta*.

[12] Knudson K. J., Price T. D., Buikstra J. E., Blom D. E. (2004). The use of strontium isotope analysis to investigate Tiwanaku migration and mortuary ritual in Bolivia and Peru. *Archaeometry*.

[13] Liu X. L., Liu C. Q., Li S. L., Li X. D. (2010). Evaluation of ground water in Liupanshui City of Guizhou Province based on the determination of *δ*
^13^C and ^87^Sr/^86^Sr. *Chinese Journal of Ecology*.

[14] Fan F. L., Fan F. Y., Qin Z. (2010). Solvent extraction of strontium with dicyclohexyl-18-crown-6. *Annual Report of the Institute of Modern Physics & Heavy Ion Research Facility of Lanzhou*.

[15] Sun A., Xu Q. C., Xu S. J., Shang G. X. H., Sun J. (2014). Ultrasonic-assisted dispersive liquid phase microextration with low-volume toxic solvent for the separation of strontium and its application for strontium isotope determination. *Asian Journal of Chemistry*.

[16] Tang S. H., Zhu X. K., Li J., Wang J. H., Yan B. (2010). Separation and isotopic measurement of Sr in rock samples using selective specific resins. *Chinese Journal of Analytical Chemistry*.

[17] Hwang E. D., Lee K. W., Choo K. H. (2002). Effect of precipitation and complexation on nanofiltration of strontium-containing nuclear wastewater. *Desalination*.

[18] Zhang N., Hu B., Huang C. Y. (2007). A new ion-imprinted silica gel sorbent for on-line selective solid-phase extraction of dysprosium(III) with detection by inductively coupled plasma-atomic emission spectrometry. *Analytica Chimica Acta*.

[19] Binder H., Zschörnig O. (2002). The effect of metal cations on the phase behavior and hydration characteristics of phospholipid membranes. *Chemistry and Physics of Lipids*.

[20] Zhao R. S., Lao W. J., Xu X. B. (2004). Headspace liquid-phase microextraction of trihalomethanes in drinking water and their gas chromatographic determination. *Talanta*.

